# Prognostic score model based on six m6A‐related autophagy genes for predicting survival in esophageal squamous cell carcinoma

**DOI:** 10.1002/jcla.24507

**Published:** 2022-05-25

**Authors:** Funan Chen, Erxiu Gong, Jun Ma, Jiehuan Lin, Canxing Wu, Shanshan Chen, Shuqiao Hu

**Affiliations:** ^1^ Department of Cardiothoracic Surgery Longyan First Hospital Longyan City China; ^2^ Priority Ward Longyan First Hospital Longyan City China

**Keywords:** autophagy, co‐expression network, m6A methylation, prognostic score

## Abstract

**Background:**

Prognostic signatures based on autophagy genes have been proposed for esophageal squamous cell carcinoma (ESCC). Autophagy genes are closely associated with m6A genes. Our purpose is to identify m6A‐related autophagy genes in ESCC and develop a survival prediction model.

**Methods:**

Differential expression analyses for m6A genes and autophagy genes were performed based on TCGA and HADd databases followed by constructing a co‐expression network. Uni‐variable Cox regression analysis was performed for m6A‐related autophagy genes. Using the optimal combination of feature genes by LASSO Cox regression model, a prognostic score (PS) model was developed and subsequently validated in an independent dataset.

**Results:**

The differential expression of 13 m6A genes and 107 autophagy genes was observed between ESCC and normal samples. The co‐expression network contained 13 m6A genes and 96 autophagy genes. Of the 12 m6A‐related autophagy genes that were significantly related to survival, DAPK2, DIRAS3, EIF2AK3, ITPR1, MAP1LC3C, and TP53 were used to construct a PS model, which split the training set into two risk groups with significant different survival ratios (*p* = 0.015, 1‐year, 3‐year, and 5‐year AUC = 0.873, 0.840, and 0.829). Consistent results of GSE53625 dataset confirmed predictive ability of the model (*p* = 0.024, 1‐year, 3‐year, and 5‐year AUC = 0.793, 0.751, and 0.744). The six‐gene PS score was an independent prognostic factor from clinical factors (HR, 2.362; 95% CI, 1.390–7.064; *p*‐value = 0.012).

**Conclusion:**

Our study recommends 6 m6A‐related autophagy genes as promising prognostic biomarkers and develops a PS model to predict survival in ESCC.

## Graphical abstract

This study aimed to explore m6A‐related autophagy genes in ESCC and develop a survival prediction model. Differential expression analysis for m6A genes and autophagy genes was performed followed by constructing a co‐expression network. Uni‐variable Cox regression analysis was performed for m6A‐related autophagy genes. Using the optimal combination of feature genes by LASSO Cox regression model, a prognostic score model was developed and subsequently validated in an independent dataset.

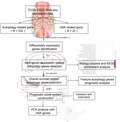

## 1 INTRODUCTION

Esophageal cancer ranks the eighth for incidence and sixth for cancer‐related mortality worldwide and is among the most aggressive human malignancies.^1^ Esophageal squamous cell carcinoma (ESCC), the dominant histological subtype in East Asian, has an extremely low five‐year survival rate and a high incidence of recurrence and metastasis.^2^ Despite recent advancements in systemic therapies of ESCC, there is a lack of approved targeted therapeutics.^3^ Discovery of prognostic biomarkers and prediction models for risk stratification holds great promise for future progress in improving patient outcomes and advancing individualized therapies in ESCC.

Autophagy, a lysosome‐dependent self‐degradation process, suppresses tumor initiation but advances tumor progression, playing opposing roles in the biology of cancer.^4^, ^5^ Past studies shows that autophagy plays a positive or negative role in regulating esophageal cell survival in a context‐dependent manner and may have important implications for patients outcomes as a promising therapeutic target.^6^, ^7^ Efficient and useful prognostic signatures based on autophagy‐related genes have been reported for predicting survival in esophageal cancer.^8^, ^9^ N6‐methyladenosine (m6A) methylation is a commonly seen modification in eukaryotic messenger RNA (mRNA) and m6A regulators are primarily composed of methyltransferases (writers), demethylases (erasers), and RNA binding proteins (readers).^10^ m6A regulators have potential as prognostic biomarkers and a strong prognostic signature based on m6A regulators has been proposed for ESCC.^11^ m6A methylation plays a crucial role in various tumorigenesis‐related biological processes, including autophagy.^12^ There is evidence that FTO, a well‐known m6A demethylase, is involved in modulating autophagosome formation in autophagy.^13^ METTL3, a primary m^6^ A methyltransferase, activates autophagy‐related pathways under hypoxia in ESCC.^14^ Moreover, two recent studies consistently show that METTL3‐mediated m6A methylation negatively modulates autophagy.^15^, ^16^ In light of the close relationships between m6A regulators and autophagy genes, we speculated that m6A‐related autophagy genes may have greater prognostic significance in ESCC.

In the present study, a comprehensive research into autophagy genes, m6A genes and their correlations in ESCC was performed. Based on the m6A‐related autophagy genes identified, a prognostic score (PS) model for survival prediction in ESCC was developed and validated. Our study might shed light on the roles of m6A‐related autophagy genes into the crucial molecular mechanisms associated with ESCC prognosis.

## 2 MATERIALS AND METHODS

### 2.1 Data acquisition

The gene expression profiles of 81 ESCC tumor samples and 11 para‐cancer tissue samples from The Cancer Genome Atlas (TCGA) data portal (https://gdc‐portal.nci.nih.gov/) was downloaded and was defined them as the training set (TCGA set) of this study. Meanwhile, GSE53625^17^, ^18^ comprising gene expression profiles of 179 pairs of ESCC tumor samples and normal tissue samples based on GPL18109 platform was downloaded from Gene Expression Omnibus (GEO) at the National Center for Biotechnology Information (NCBI, https://www.ncbi.nlm.nih.gov/) and used as a validation set.

### 2.2 Identification and function annotation of m6A‐related autophagy genes

Total 232 autophagy genes were firstly downloaded from HADb (Human Autophagy Database, http://www.autophagy.lu/) and expression data of 22 m6A genes (METTL3, METTL14, METTL15, WTAP, VIRMA, RBM15, RBM15B, KIAA1429, ZC3H13, FTO, ALKBH5, RBMX, YTHDC1, YTHDC2, IGF2BP1, IGF2BP2, IGF2BP3, YTHDF1, YTHDF2, YTHDF3, HNRNPA2B1, and HNRNPC) was extracted from the training set. The differential expression analysis of these autophagy genes and m6A genes was performed between tumor and normal samples using student's *t* test in R (version 3.6.1), with a significance threshold of *p*‐value <0.05.

Relationships between differentially expressed autophagy genes and m6A regulatory genes (*p*‐value <0.05) were evaluated by calculating their Pearson correlation coefficients (PCCs) with cor function (http://77.66.12.57/R‐help/cor.test.html) in R. The significant gene pairs with PCC > 0.4 and *p*‐value <0.05 was selected to construct an m6A‐autophagy genes co‐expression network, followed by visualization using Cytoscape^19^ software (version 3.6.1, https://cytoscape.org/). Gene ontology (GO) and Kyoto Encyclopedia of Genes and Genomes (KEGG) pathway enrichment analyses^7^ were performed for all genes in the co‐expression network using Database for Annotation Visualization and Integrated Discovery (DAVID)^20^ bioinformatics resources (version 6.8, https://david.ncifcrf.gov/). Significance was defined as false discovery rate (FDR) < 0.05.

### 2.3 Development and validation of PS model based on feature autophagy genes

The uni‐variate Cox regression analysis was performed for the data in training set to select the autophagy genes that were significantly related to overall survival (OS) time from the autophagy genes in the co‐expression network using survival package (version 2.41–1, http://bioconductor.org/packages/survivalr/) in R. *p*‐Value<0.05 was regarded as a significance threshold.

Least absolute shrinkage and selection operator (LASSO) Cox regression model^21^ along with 1000‐fold cross‐validation likelihood was employed to identify the optimal combination of feature autophagy genes using penalized package (https://cran.r‐project.org/web/packages/penalized/index.html) in R.^22^ We put data of the significant survival‐related genes in LASSO Cox regression model to achieve variable selection and shrinkage. Cross‐validation (CV) procedure was iterated for 1000 times to determine the optimal penalty regularization parameter λ.

We further explored associations of each feature autophagy gene with OS of patients in the training set using survival package. According to median expression level of each feature autophagy gene, all samples in the training set were divided into high expression group (expression value was higher than the median expression level) and low expression group (expression value was lower than the median expression level), separately.

Based on expression of feature autophagy genes weighted by LASSO Cox regression coefficients, PS was calculated for each sample as follows:

$$\text{Prognostic score}\;\left(\mathrm{PS}\right)=\sum \beta_{\text{genes}}\times {\mathrm{Exp}}_{\text{genes}}$$

Wherein β_genes_ denotes LASSO Cox regression coefficient, and Exp_genes_ denotes gene expression level in the training set.

According to median PS value in the training set or the validation set, samples were split into a high‐risk subgroup (PS was higher than the median PS) and low‐risk subgroup (PS was lower than the median PS). Survival analysis was carried out using survival package with plotting Kaplan–Meier curves. OS time of different groups was compared using log‐rank test. The area under the ROC curve (AUC) is used to assess predictive performance of the PS model. Uni‐ and multi‐variable Cox regression analyses were performed to identify independent prognostic factors using survival package. Log‐rank *p* < 0.05 suggested significance.

### 2.4 Principal component analysis

Based on expression levels of the m6A genes, principal component analysis (PCA), a dimension reduction technique,^23^, ^24^ was used to classify samples in the training set and the validation set, respectively, using psych package (version 1.7.8, https://cran.r‐project.org/web/packages/psych/index.html) in R.

## 3 RESULTS

### 3.1 A co‐expression network composed of 13 m6A genes and 107 autophagy genes was constructed

**FIGURE 1:**
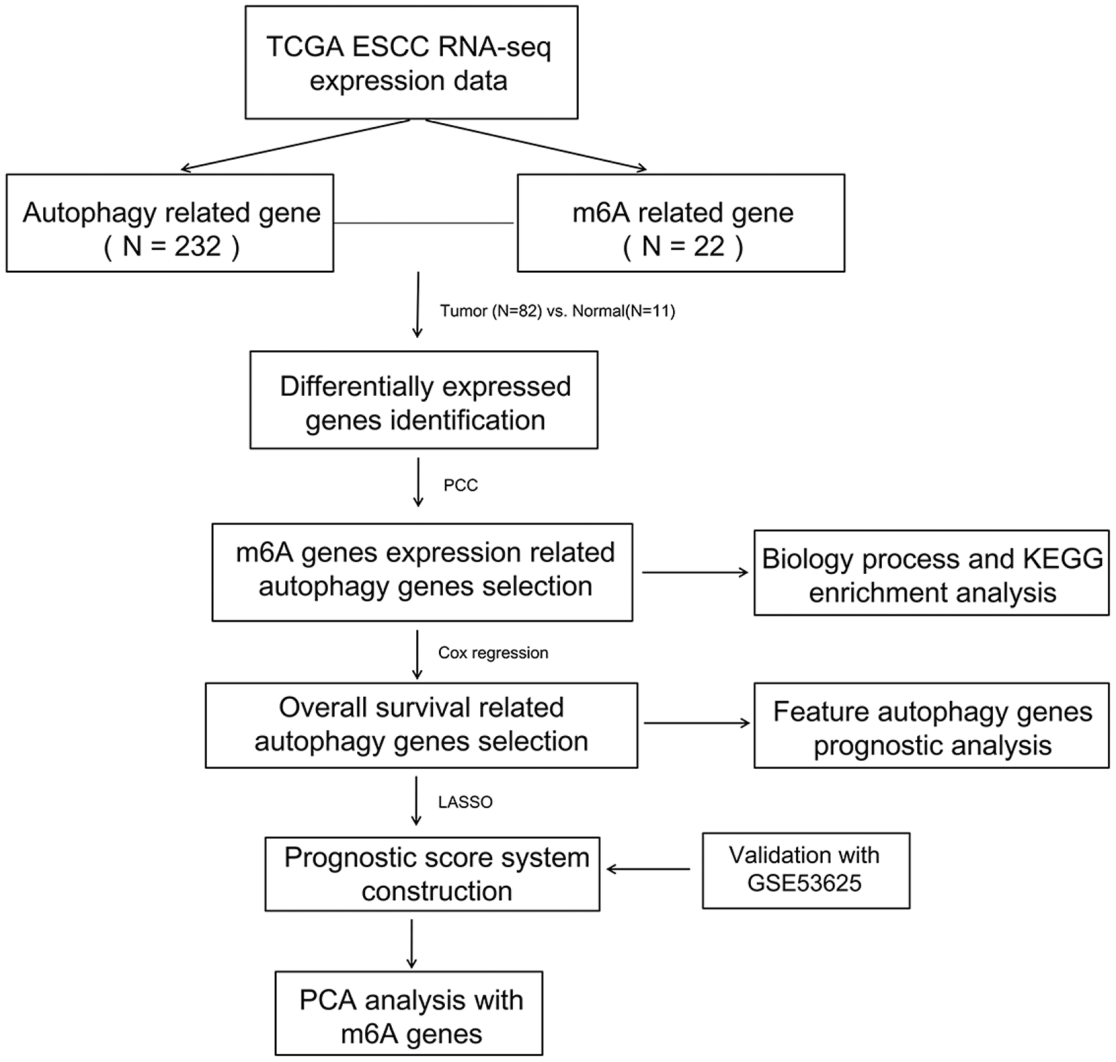
Flowchart of overall study design

**FIGURE 2:**
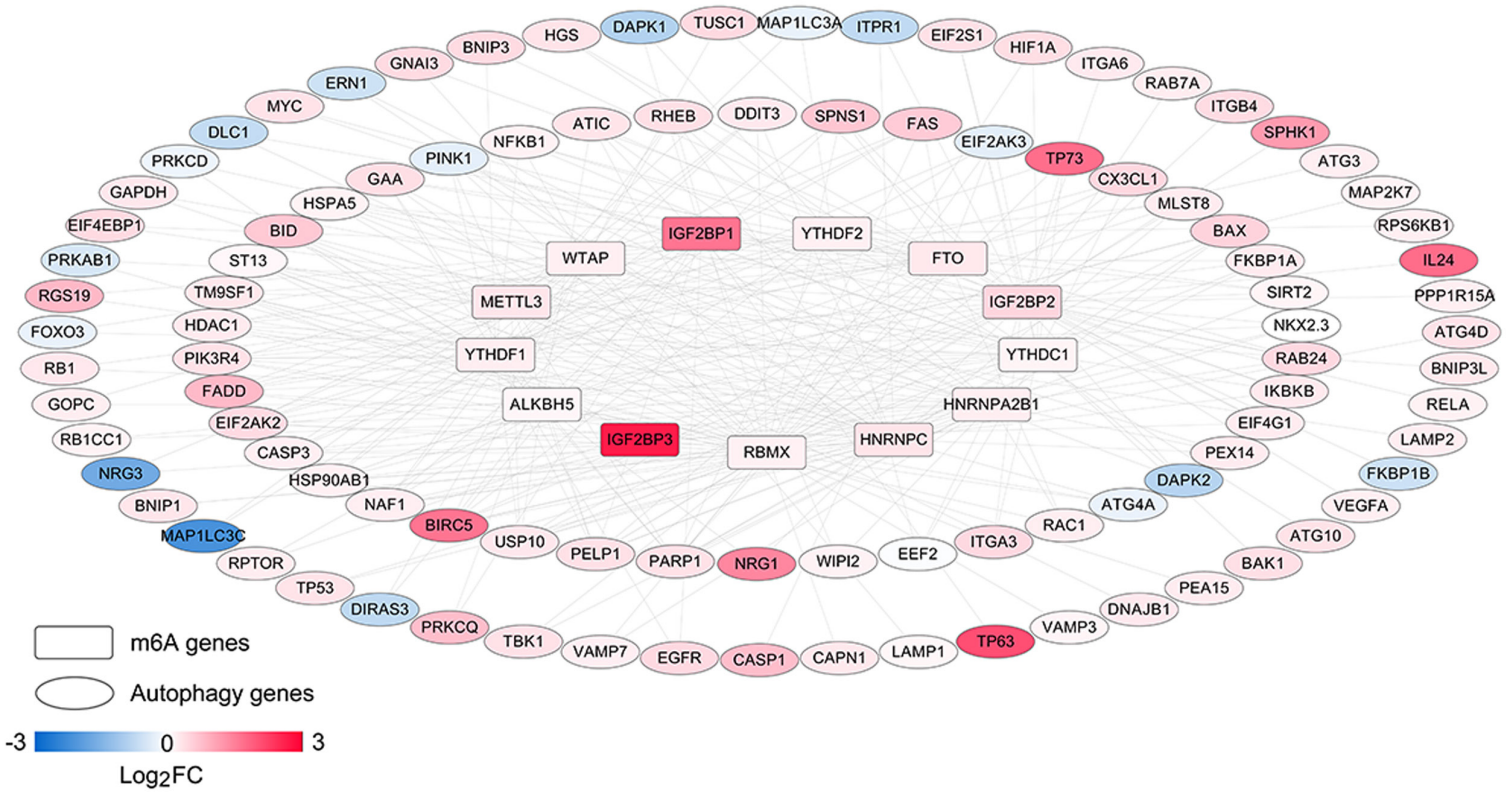
A m6A‐autophagy genes co‐expression network. There are 13 m6A genes and 96 autophagy genes in the network. Rectangle and oval nodes represent m6A genes and autophagy genes, respectively. Links between two nodes represent correlations between two genes. Color bar from blue to red suggests log_2_FC value from −3 to 3

Flowchart of data analysis is shown in Figure 1. We identified 13 m6A genes and 107 autophagy genes whose expression levels were significantly different (*p*‐value <0.05) between tumor samples and normal samples in the training set. Using their expression data, we obtained 304 co‐expression gene pairs with PCC > 0.4 and *p*‐value <0.05. Accordingly, a co‐expression network including 13 m6A genes and 96 autophagy genes was constructed (Figure 2).

**FIGURE 3:**
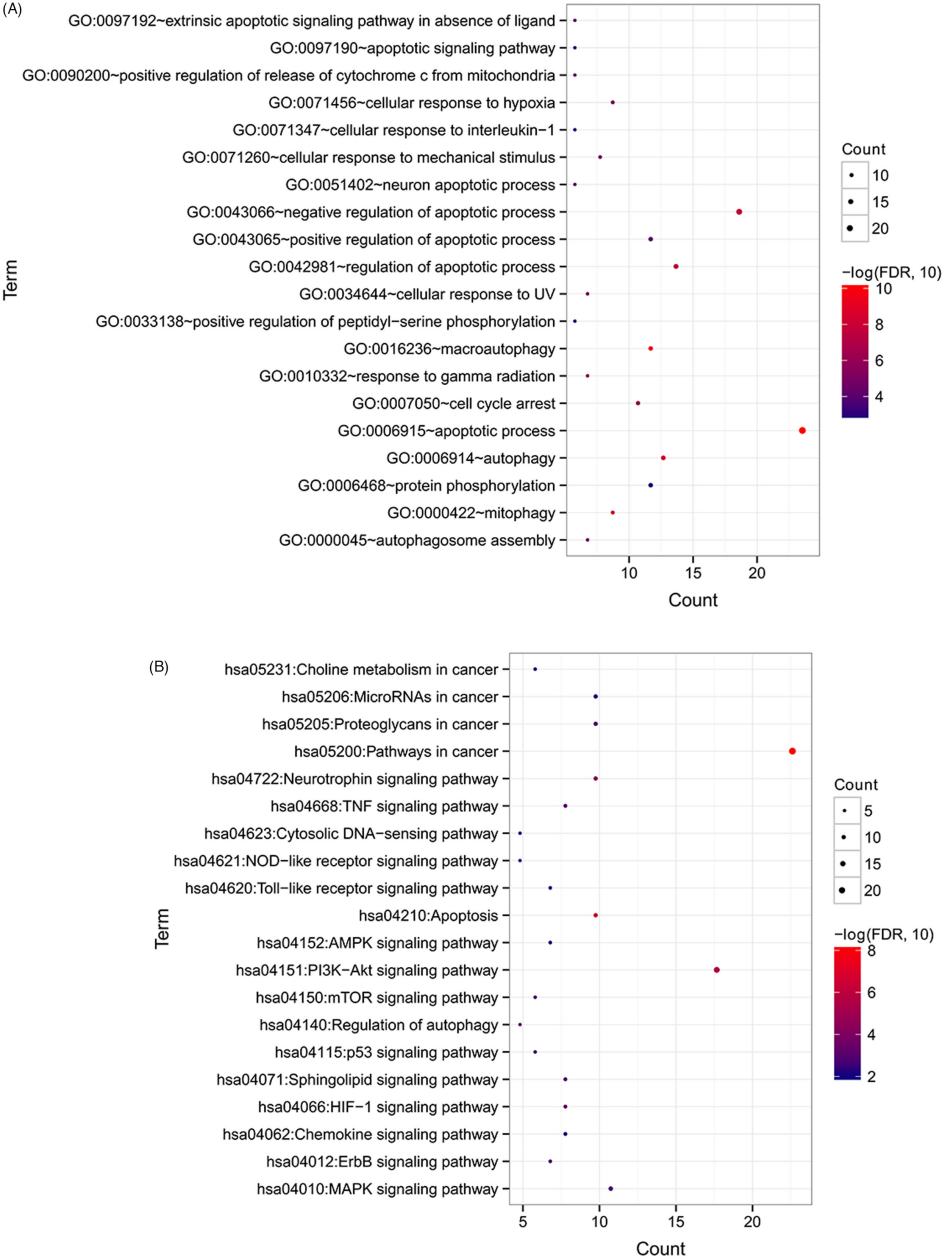
GO function and KEGG pathway enrichment analysis. (A) Significant biological processes identified by GO enrichment analysis. (B) Significant signaling pathways identified by KEGG pathway enrichment analysis. Count denotes the number of significantly enriched genes involved in a GO biological process or a signaling way. Size of round nodes is positively correlated with count of genes

GO and KEGG pathway enrichment analyses for the autophagy genes in the co‐expression network revealed a significant enrichment of 50 biological processes and 25 KEGG signaling pathways. According to FDR, top 20 biological processes and KEGG signaling pathways are selected and listed in Figure 3, such as several apoptosis or autophagy‐related biological processes, NOD‐like receptor signaling pathway, Toll‐like receptor signaling pathway, PI_3_K‐Akt signaling pathway, and MAPK signaling pathway.

### 3.2 A PS model based on 6 feature autophagy genes for risk stratification in ESCC

Among the 96 autophagy genes in the co‐expression network, 12 genes were significantly associated with the OS time of patients in the training set in uni‐variate Cox regression analysis. The 12 survival‐related genes were further used as input for LASSO Cox regression model. When mean squared error (1se) = 0.07874, an optimal combination of 6 feature autophagy genes was obtained (Figure S1), including DAPK2, DIRAS3, EIF2AK3, ITPR1, MAP1LC3C, and TP53.

**FIGURE 4:**
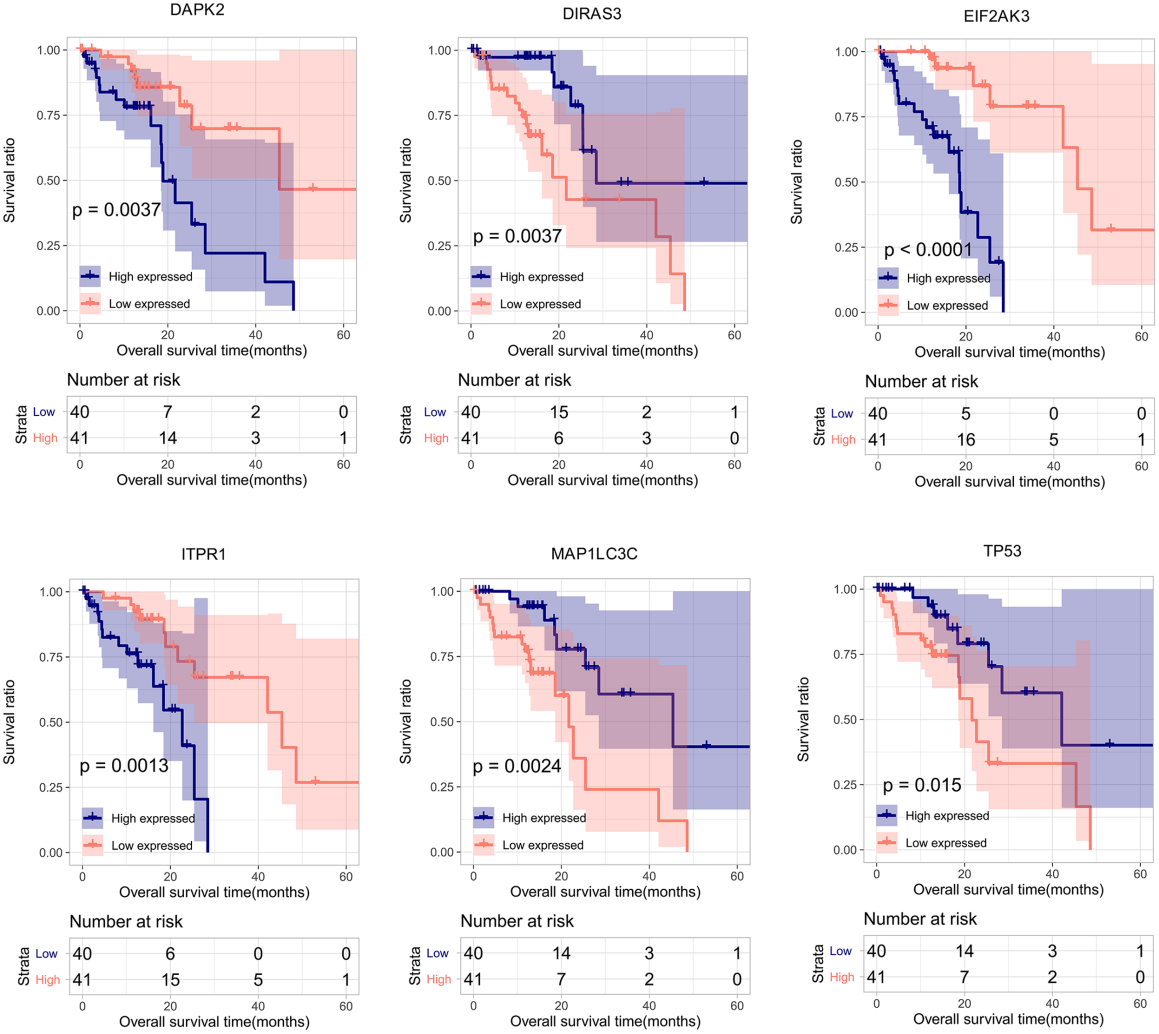
Kaplan–Meier overall survival curves for the high expression group and the low expression group. All patients are separated into a high expression group and a low expression group according to median expression level of DAPK2, DIRAS3, EIF2AK3, ITPR1, MAP1LC3C, or TP53

According to the median expression level of each feature autophagy gene, samples in the training set were classified into a high expression group and a low expression group, separately. As shown in Kaplan–Meier curves in Figure 4, the OS time of the patients in the high expression group was significantly different than that in low expression group (DAPK2, *p* = 0.0037; DIRAS3, *p* = 0.0037; EIF2AK3, *p* < 0.0001; ITPR1, *p* = 0.0013; MAP1LC3C, *p* = 0.0024; TP53, *p* = 0.015), suggesting that expression levels of the six autophagy genes were closely related to survival of ESCC patients.

Based on LASSO Cox regression coefficients and expression levels of the 6 feature autophagy genes, PS was calculated for each sample using the following formula:

$$\begin{array}{c} \mathrm{PS}=\left(-0.05391685\right)\times \mathrm{Exp}\;\text{DAPK2}+\left(0.09794721\right)\times \mathrm{Exp}\;\text{DIRAS3}+\left(-0.11247539\right)\times \mathrm{Exp}\;\mathrm{EIF}2\text{AK3}\\ \;+\left(-0.08958837\right)\times \mathrm{Exp}\;\text{ITPR1}+\left(0.28225026\right)\times \mathrm{Exp}\;\mathrm{MAP}1\mathrm{LC}3C+\left(0.01569142\right)\times \mathrm{Exp}\;\mathrm{TP}53. \end{array}$$

**FIGURE 5:**
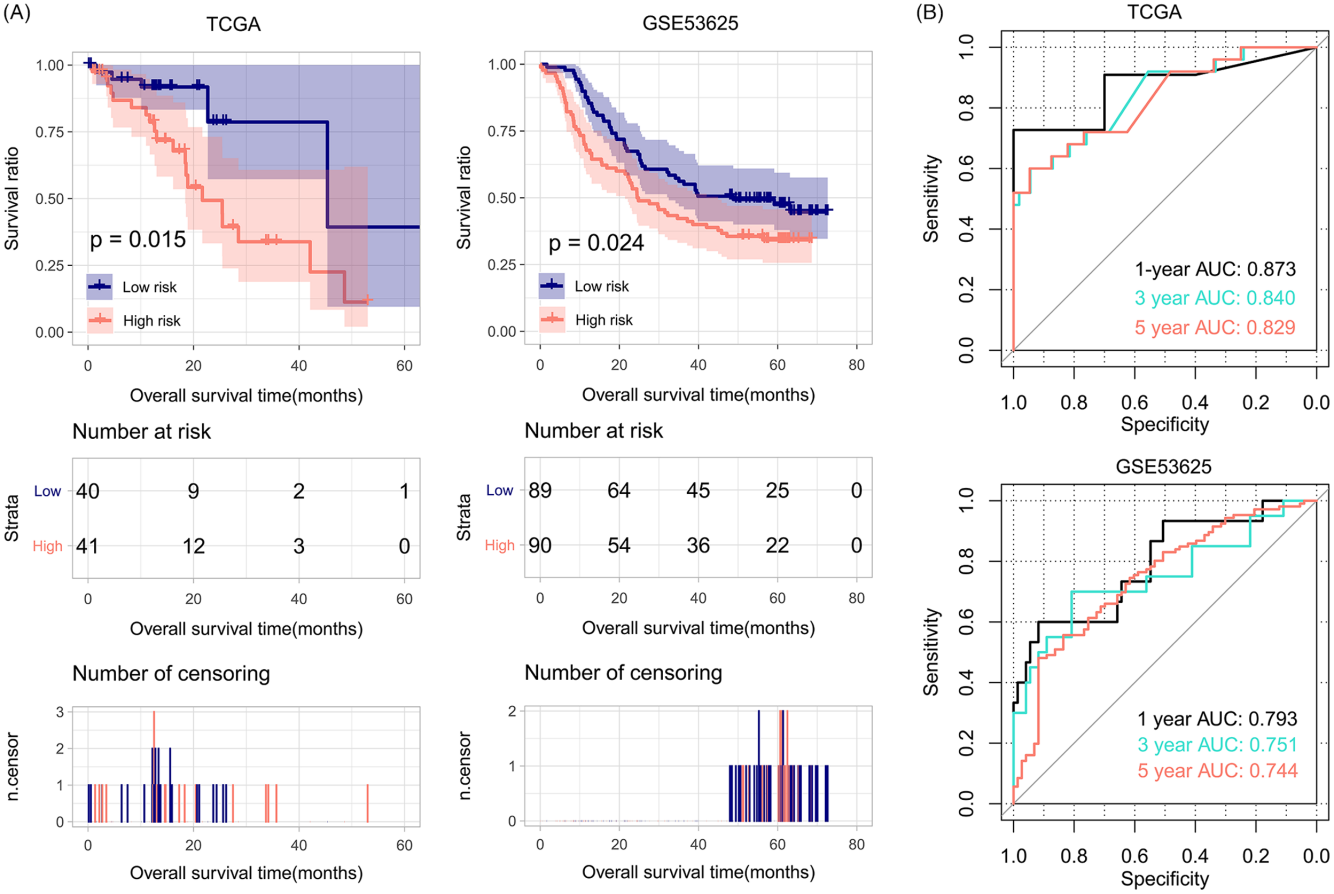
Kaplan–Meier overall survival curves (A) and ROC curves (B) for the TCGA set and GSE53625. Patients are divided into a high‐risk group and a low‐risk group by the six‐gene PS model. The high‐risk group has significantly lower survival ratio compared to the low‐risk group (*p* = 0.015, 0.024)

Using median PS as cutoff, the training set was separated into a high‐risk group and a low‐risk group with significantly different OS time (*p* = 0.015, Figure 5A). Besides, 1‐, 3‐, and 5‐year AUC were 0.873, 0.840, and 0.829, separately (Figure 5B).

### 3.3 Successful validation of the PS model in GSE53625 dataset

Prognostic score model based on the 6 feature autophagy genes was further tested on an independent validation set of GSE53625 to verify its prognostic capability in ESCC. The high‐risk group had significantly lower survival ratio compared with the low‐risk group (*p*‐value = 0.024, Figure 5A), similar to the results of the training set. Figure 5B shows that 1‐year, 3‐year, and 5‐year AUC were 0.793, 0.751, and 0.744. These results proved that PS based on the six feature autophagy genes was efficient and accurate in discriminating high‐risk patients from low‐risk patients in ESCC.

As shown in Table 1, the six‐gene PS model (*p*‐value = 1.495E‐02), sex (*p*‐value = 1.841E‐03), pathologic N (*p*‐value = 9.377E‐03), and pathologic stage (*p*‐value = 4.585E‐02) were statistically significant in uni‐variate Cox regression analysis. Furthermore, multi‐variable Cox regression analysis suggested only the six‐gene PS model was an independent prognostic factor (HR, 2.362; 95% CI, 1.390–7.064; *p*‐value = 1.242E‐02) for predicting the prognosis of ESCC.

**TABLE 1:** Analysis of prognostic clinical factors

Clinical characteristics	Uni‐variable Cox		Multi‐variable Cox	
	HR [95% CI]	*p* Value	HR [95% CI]	*p* Value
Age (years, mean ± SD)	1.034 [0.992–1.078]	1.106E‐01	—	—
Sex (Male/Female)	9.833 [1.294–74.73]	1.841E‐03	7.889 [0.938–67.08]	5.860E‐02
Neoplasm histologic grade (G1/G2/G3)	1.087 [0.582–2.208]	7.941E‐01	—	—
Pathologic M (M0/M1)	3.197 [0.909–11.24]	7.002E‐02	—	—
Pathologic N (N0/N1/N2/N3)	1.915 [1.160–3.163]	9.377E‐03	1.392 [0.707–2.738]	3.387E‐01
Pathologic T (T1/T2/T3/T4)	1.095 [0.636–1.886]	7.432E‐01	—	—
Pathologic stage(I/II/III/IV)	1.728 [1.010–2.956]	4.584E‐02	0.899 [0.386–1.855]	6.760E‐01
Tumor recurrence (Yes/No/−)	1.031 [0.315–3.372]	9.601E‐01	—	—
Alcohol history (Yes/No)	2.962 [0.690–12.71]	1.439E‐01	—	—
PS status (High/Low)	3.203 [1.192–8.609]	1.495E‐02	2.362 [1.390–7.064]	1.241E‐02

Abbreviations: CI, confidence interval; HR, hazard ratio; PS, prognostic score; SD, standard deviation.

### 3.4 PCA analysis for high‐risk and low‐risk samples based on m6A genes

**FIGURE 6:**
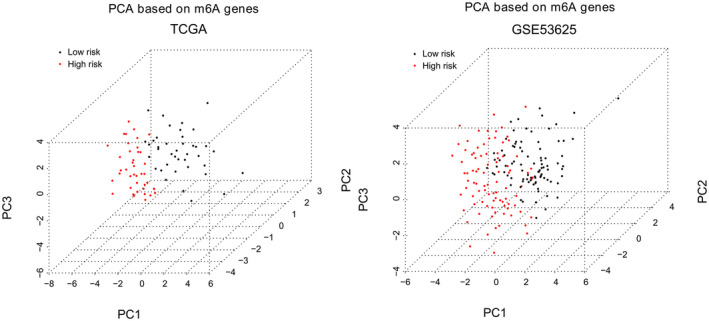
PCA maps show separation of high‐risk patients from low‐risk patients based on m6A genes in the TCGA set and GSE53625 dataset

**FIGURE 7:**
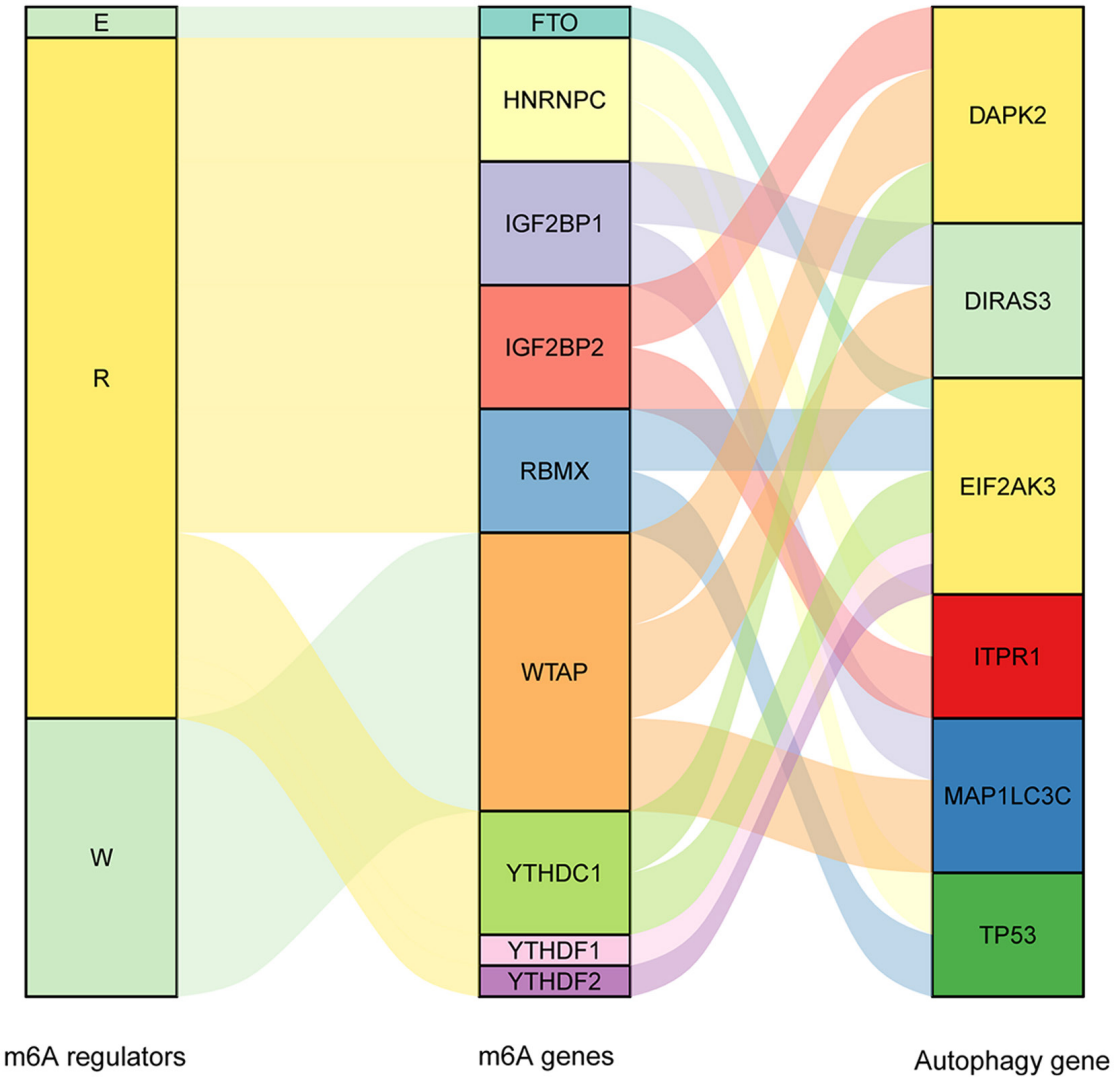
Schematic illustration of correlations between m6A genes, m6A regulators, and autophagy genes in Sankey charts

Principal component analysis analysis was further performed to classify samples based on expression levels of m6A genes identified in the training set. Using PC1, PC2, and PC3 as depicted in Figure 6, three‐dimensional map showed a clear separation of high‐risk samples from low‐risk samples based on expression levels of m6A genes in the training set (TCGA set) and the validation set (GSE53625). Sankey chart in Figure 7 shows that the 6 feature autophagy genes (DAPK2, DIRAS3, EIF2AK3, ITPR1, MAP1LC3C, and TP53) were connected with m6A genes that belong to three types of m6A regulators including writers, erasers, and readers. These are indirect evidences for prognostic significance of the 6 feature autophagy genes in ESCC.

## 4 DISCUSSION

m6A RNA methylation regulators play a role in proliferation and migration of ESCC cells and are associated with prognosis of ESCC patients.^11^, ^25^ Nevertheless, little is known concerning biological roles of m6A‐related autophagy genes in ESCC progression and patient prognosis. In the current study, we mined differentially expressed m6A genes and autophagy genes in ESCC and constructed an m6A‐autophagy genes co‐expression network. Twelve m6A‐related autophagy genes were found to be independent predictive factors, and a prognostic signature of the 6 feature autophagy genes was established. Moreover, our study developed and validated the PS model based on the 6 feature autophagy genes for predicting survival of ESCC patients. The present study broadened our knowledge regarding relationships between m6A genes and autophagy genes as well as prognostic implications of m6A‐related autophagy genes in ESCC.

One important innovation of our study is an m6A‐autophagy genes co‐expression network of 13 m6A genes and 96 autophagy genes. These autophagy genes in the co‐expression network were closely related to m6A genes and significantly involved in various apoptosis or autophagy‐related biological processes and diverse signaling pathways, such as NOD‐like receptor signaling pathway, Toll‐like receptor signaling pathway, PI_3_K‐Akt signaling pathway, mTOR signaling pathway, and MAPK signaling pathway. NOD‐like receptor signaling pathway and Toll‐like receptor signaling pathway are major regulators of cancer‐related inflammation and immunity.^26^, ^27^ PI_3_K‐Akt signaling pathway, MAPK signaling pathway, and mTOR signaling pathway are well‐documented players in cell proliferation and migration of ESCC.^28^, ^29^, ^30^ Additionally, mTOR signaling pathway participates in regulating autophagy.^31^ These results contribute to a deeper understanding of biological functions of m6A‐related autophagy genes in ESCC.

Past studies lay emphasis on prognostic signatures and models based on autophagy genes^8^ or m6A genes.^11^ Our PS model was based on the expression of 6 feature autophagy genes closely related to m6A genes and could differentiate high‐risk ESCC patients from low‐risk ESCC patients with significantly different OS time in the training set. Moreover, predictive ability of the model was successfully validated in an independent set. Notably, our model yielded higher AUC values for the training set (0.873) and the validation set (0.793) than the prognostic models based on autophagy genes (0.746, 0.691)^8^ or m6A genes (0.73, 0.6),^11^ suggesting superior predictive ability of our model to the other models. Furthermore, uni‐ and multi‐variable Cox regression analyses suggest that the six‐gene PS model could serve as an independent prognostic factor for predicting prognosis of ESCC.

The 6 feature autophagy genes in PS model were DAPK2, DIRAS3, EIF2AK3, ITPR1, MAP1LC3C, and TP53. DIRAS3 (DIRAS family GTPase 3), namely ARHI, is an imprinted anti‐oncogene and a negative prognostic biomarker in glioblastoma multiforme.^32^ DIRAS3 overexpression inhibits cell proliferation, promotes apoptosis and autophagy, and may serve as a prognostic biomarker and candidate therapeutic target in ESCC.^33^ EIF2AK3 (eukaryotic translation initiation factor 2 alpha kinase 3), also called protein kinase PERK, is a transducer of unfolded protein responses implicated in endoplasmic reticulum stress, contributing to cancer development.^34^ EIF2AK3 participates in modulating cell growth, colony formation and apoptosis and may affect clinical prognosis in ESCC.^35^ TP53 (tumor protein 53) is the most recurrently mutated gene in ESCC. Accumulating studies have shown that TP53 mutation has prognostic value for several cancers, such as hepatocellular carcinoma^36^ and ESCC.^37^ Our study showed that TP53 is significantly enriched in PI_3_K‐Akt signaling pathway and MAPK signaling pathway, which was in line with previous demonstration.^38^ DAPK2, (death‐associated protein kinase 2), a Ca^2+^/calmodulin‐regulated serine/threonine kinase, has been characterized as a critical regulator of apoptosis, autophagy, and inflammation and a tumor suppressor.^39^, ^40^ DAPK2 is involved in regulating oxidative stress in cancer cells via MAPK pathway.^41^ ITPR1 (inositol 1,4,5‐trisphosphate receptor 1) is a ligand‐gated ion channel in regulating calcium release from endoplasmic reticulum and acts as a autophagy sensor.^42^ ITPR1 down‐regulation is observed in esophageal adenocarcinoma and has been recognized as a potential biomarker of prognosis in esophageal adenocarcinoma.^43^, ^44^ MAP1LC3C (microtubule‐associated protein 1 light chain 3 gamma), a critical structural protein in autophagosome membrane, has been reported to be an independent prognostic biomarker in colorectal cancer.^45^, ^46^ However, there is little knowledge concerning prognostic implications of DAPK2, ITPR1, and MAP1LC3C in ESCC. Our study suggested the 6 feature autophagy genes as potential prognostic biomarkers in ESCC. Additionally, PCA analysis showed evident differentiation of high‐risk patients from low‐risk patients based on expression levels of the 6 feature autophagy genes, further confirming their prognostic value.

Our PS model of 6 feature autophagy genes might offer a new strategy for risk stratification and prognosis assessment in ESCC. For a cohort of ESCC patients or people who are healthy but have a familial background for ESCC, the expression levels of the 6 feature autophagy genes in the PS model could be measured by laboratory analysis, such as quantitative reverse transcriptase‐polymerase chain reaction. Then, the PS could be calculated by the formula in our study, and the people could be assigned into high‐risk or low‐risk group to predict their prognosis. Because of the relative high‐AUC values of this PS model, we anticipate it could increase the prognosis predicting accuracy of ESCC patients in clinic in the future.

Nevertheless, some drawbacks should be acknowledged. Firstly, sample size of the training set is limited, and more validation sets are required. Secondly, experimental validations should be conducted in future studies. Thirdly, more efforts are necessary to uncover biological functions of these m6A‐related autophagy genes. Fourthly, nomogram incorporating the six‐gene PS and well‐defined prognostic clinical factors should be considered to improve its predictive performance.

## 5 CONCLUSION

Combining data from TCGA and HADb databases with comprehensive bioinformatics analysis, we unraveled a list of m6A‐related autophagy genes in ESCC. We identified 6 promising prognostic genes, developed, and validated a PS model based on them, which performed well in distinguishing high‐risk patients from low‐risk patients in ESCC. This study provides a more detailed portrait of m6A genes and autophagy genes in the biology of ESCC and facilities individualized outcome prediction for patients.

## CONFLICT OF INTEREST

The authors declare that they have no conflict of interest.

## Supporting information


Figure S1

## Data Availability

Gene expression data analyzed in this study are available from The Cancer Genome Atlas (TCGA) data portal (https://gdc‐portal.nci.nih.gov/) and Gene Expression Omnibus (GEO) at the National Center for Biotechnology Information (NCBI, https://www.ncbi.nlm.nih.gov/) with accession number of GSE53625.
