# Supplementary figures and images for: Prognostic score model based on six m6A‐related autophagy genes for predicting survival in esophageal squamous cell carcinoma
Source: J Clin Lab Anal. 2022 May 25;36(7):e24507. doi: 10.1002/jcla.24507 (PMC9279981; doi:10.1002/jcla.24507)

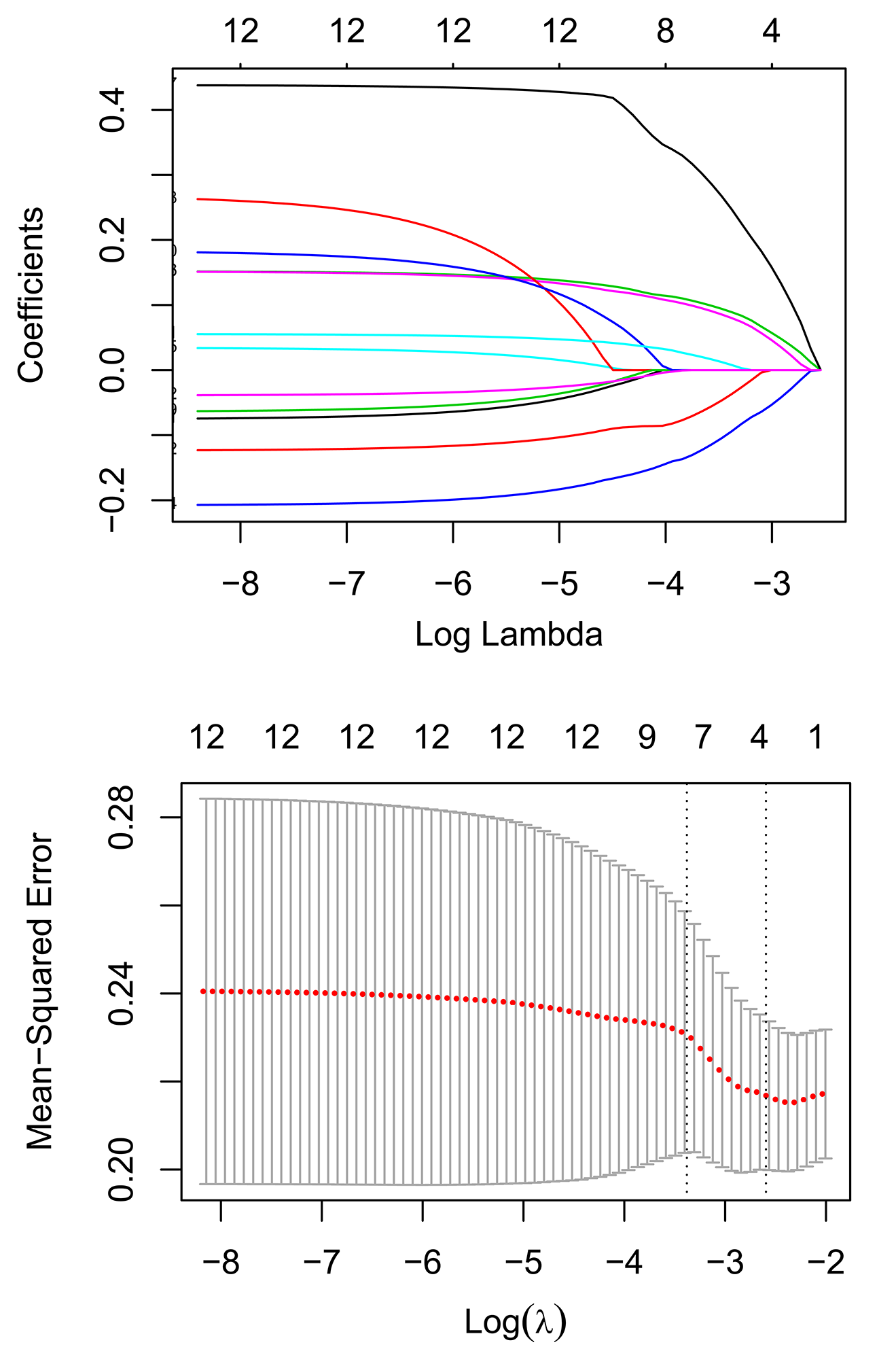

Supplement: Supplementary file 1 — Figure S1 [file JCLA-36-e24507-s001.tif]
